# Urogenital chlamydia trachomatis treatment failure with azithromycin: A meta-analysis

**DOI:** 10.18502/ijrm.v17i9.5093

**Published:** 2019-09-22

**Authors:** Farnaz Mohammadzadeh, Mahrokh Dolatian, Masoumeh Jorjani, Maryam Afrakhteh, Hamid Alavi Majd, Fatemeh Abdi, Reza Pakzad

**Affiliations:** ^1^ Department of Midwifery and Reproductive Health School of Nursing and Midwifery Shahid Beheshti University of Medical Sciences Tehran Iran.; ^2^ Department of Pharmacology School of Medicine Shahid Beheshti University of Medical Sciences Tehran Iran.; ^3^ Department of Obstetrics and Gynaecology Tajrish Shohada Shahid Beheshti University of Medical Sciences Tehran Iran.; ^4^ Department of Biostatistics Paramedical School Shahid Beheshti University of Medical Sciences Tehran Iran.; ^5^ Social Determinants of Health Research Center Alborz University of Medical Sciences Karaj Iran; ^6^ Noor Research Center for Ophthalmic Epidemiology Noor Eye Hospital Tehran Iran.; ^7^ Faculty of Health Ilam University of Medical Sciences Ilam Iran

**Keywords:** Azithromycin, Chlamydia trachomatis, Urogenital, Treatment failure, Meta-analysis

## Abstract

**Background:**

Chlamydia Trachomatis is one of the most common pathogens transmitted through the genital tract in humans that leads to urogenital infection.

**Objective:**

Given the high prevalence of chlamydia infection and its adverse effects on the health of women and men, the present meta-analysis was conducted to determine the rate of treatment failure with azithromycin.

**Materials and Methods:**

Databases including MEDLINE, ISI - Web of Science, PubMed, EMBASE, Scopus, ProQuest, and Science Direct were searched for articles published between 1991 and 2018. The quality of the selected articles was assessed using the Cochrane risk of bias assessment tool. Heterogeneity was determined using the I2 and Cochrane Q-Test. Subgroup analysis and meta-regression were used to compare the prevalence rates on different levels of the variables.

**Results:**

A total of 21 articles that met the inclusion criteria were ultimately assessed. The pooled estimate of azithromycin failure rate was 11.23% (CI 95%: 8.23%-14.24%). Also, the azithromycin failure rate was 15.87% (CI 95%: 10.20%-21.54%) for the treatment of urethritis, 7.41% (CI 95%: 0.60%-14.22%) for cervicitis, and 7.14% (CI 95%: 10.90%-3.39%) for genital chlamydia. The pooled estimate of failure rate difference was 2.37% (CI 95%: 0.68%-4.06%), which shows that azithromycin has a higher failure rate in the treatment of chlamydia compared to doxycycline and other examined medications. The meta-regression results showed that the patient's age contributes significantly to the heterogeneity for azithromycin treatment failure rate (*β*░=░0.826; p░=░0.017).

**Conclusion:**

Azithromycin has a higher failure rate than doxycycline and other studied medications in treating urogenital chlamydia infections.

## 1 Introduction

Chlamydia Trachomatis is an intracellular gram-negative bacterium and one of the most common pathogens transmitted through the genital tract in humans and causing urogenital infection. Chlamydia is one of the most common Sexually Transmitted Diseases (STDs) in the world, which has a higher prevalence among adolescents and women (1). According to the latest World Health Organization (WHO) reports, the reported prevalence of chlamydia has steadily increased over the last two decades, and 92 million new cases of chlamydia trachomatis infection occurred in 2009, reaching 101, 131, and 153 million in 2011, 2012, and 2015, respectively (2,3). The risk factors for chlamydial infection include age 15–24, several sexual partners, unprotected sex, and previous history of STDs. Being single and a poor socioeconomic status and education level is also associated with the incidence of this disease (4,5).

The results from previous studies suggest that chlamydia trachomatis infection entails the risk of cervicitis, urinary tract infections, prostatitis, epididymitis, peritonitis, lower respiratory tract diseases, chronic fatigue syndrome, and reactive arthritis. It also doubles the risk of ectopic pregnancy (6,7) and increases the risk of developing the inflammatory pelvic disease in recurring chlamydial infections or treatment failures by 4–6 times (8). Chlamydia infection also increases the likelihood of Human Immunodeficiency Virus (HIV) comorbidity. Infertility is one of the common complications of untreated chlamydial cervicitis, whose likelihood increases with the frequency of recurrence or treatment failure (9,10). The proposed treatment of Chlamydial cervicitis is a single dose of oral azithromycin or oral doxycycline 100░mg twice daily for seven days. Alternative therapies include oral tetracycline 500░mg four times daily for seven days, oral erythromycin 500░mg four times daily for seven days, and oral ofloxacin 200░mg twice daily for seven days. According to the latest WHO guidelines, the evidence derived from comparing different chlamydial infection treatment regimens is poor to moderate, and further controlled, randomized, trials comparing these treatments and recommended doses are necessary (11).

Azithromycin treatment failure was first reported in Australia and subsequently documented in several continents. The common causes of treatment failure include bacterial resistance to azithromycin, improper absorption of azithromycin by the upper vagina, and the ineffective antibiotic coverage of this routine treatment on certain common pathogenic bacteria associated with chlamydia infection. Recent studies have shown a prevalence of 41.4% (range: 17.7% to 56.6%) for mutations related to the resistance of Chlamydiatrachomatis strains against azithromycin (5,12–14). Since the failure to choose properly covering antibiotics and frequent treatment failures for chlamydial cervicitis cause incurable infections that entail a risk of inflammatory pelvic disease, urinary tract infections, peritonitis, ectopic pregnancy, premature rupture of the amniotic sac and infertility, further clinical studies are required to determine the right treatment program and medication dosage for an effective clinical treatment (15–19).

Studies show contradictory information regarding treatment failure with azithromycin for chlamydial infection compared to other treatment strategies. Since systematic review studies summarize the reported results and provide evidence in the best way and with great clarity (20), the present meta-analysis study was conducted to determine the rate of treatment failure with azithromycin in patients with urogenital chlamydial infection.

## 2 Materials and Methods

### 2.1 Search strategy

The preferred reporting items for systematic reviews and meta-analyses (PRISMA) guidelines were followed to report the findings of this study. Studies published before August 2018 were identified without any language restrictions through an electronic search of databases including MEDLINE, ISI - Web of Science, PubMed, EMBASE, Scopus, ProQuest, and Science Direct. A combination of keywords including “Azithromycin” or “AZT” or "Single-dose Azithromycin" and “Chlamydia trachomatis” or “Chlamydia” or “Genital chlamydia infection” or“Urethritis” or “Cervicitis” or "Endocervicitis" or “Cervicovaginitis” or “Urogenital” or “Rectal chlamydia”, and “Treatment failure” or “failure” were used to collect data.

### 2.2 Eligibility criteria

Trials comparing azithromycin with doxycycline or other medications for the treatment of Chlamydiatrachomatis in women (cervicitis, urethritis, and rectal infection) or men (urethritis and rectal infection) were assessed. The inclusion criteria were: (1) Being a randomized clinical trial or a clinical trial; (2) Comparing treatment regimens of oral azithromycin (single- or multiple-dose regimens) with oral doxycycline (twice daily 100░mg for seven days) or other regimens; (3) Being a man or woman over the age of 12; (4) Assessment of microbial or clinical recovery during a follow-up period of 1–9 wk; and (5) Reporting treatment failure during the trial.

The articles that had published only their abstract, the in vitro or in vivo studies, anything other than Randomized Controlled Trials (RCTs) or clinical trials, and articles that did not provide the numerical outcome data were eliminated.

### 2.3 Study selection

**Figure 1:**
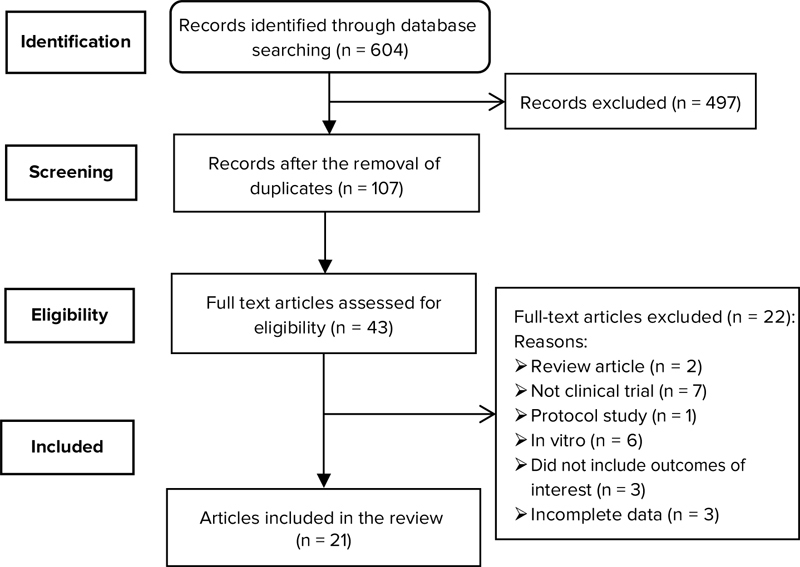
Flowchart showing the selection of studies.

The eligibility of the articles was assessed independently by two authors, and any disagreements were resolved by consensus. A total of 604 irrelevant or duplicate articles were eliminated in this stage. The assessment of the articles and abstracts led to the exclusion of another 497 articles. In assessing the full texts, 22 articles out of the remaining 43 did not meet the eligibility criteria and were excluded. A total of 21 eligible articles were ultimately assessed (Table I and Figure 1).

**Table I:** The characteristics of the studies included in the systematic review

Author (year) ref no.	Country	Study type	Age	Participants	Chief complain	Diagnostic method	Intervention		Comparison		Follow up (week)	Treatment failure (%)
Geisler (2015) (22)	USA	RCT	Median: 17	567 males and females	Genital chlamydia	NAAT	AZT 1░g oral single dose (n░=░284)		Doxycycline 100░mg oral BID for 7 days (n░=░283)		4	AZT: 3.2 Doxycycline: 0
Geisler (2014) (23)	USA	RCT	Mean: 23.9 (4.6)	82 female	Genital chlamydia	NAAT	AZT 1░g oral single dose (n░=░42)		Rifalazil 25░mg oral single dose (n░=░40)		3–4, 5–6	AZT: 7.9 Rifalazil: 15.2
Beyda (2014),(24)	USA	CT	Mean: 15.6 (0.86)	128 male and female	Genital chlamydia	NAAT	AZT 1░g oral single dose		Without comparison group		3–4	AZT: 3.9
Takahashi (2014) (25)	Japan	CT	Median: 33	200 male	Urethritis	Symptoms and NAAT	AZT 2░g oral single dose (n░=░200)		Without comparison group		1–4	AZT: 9.09
Manhart (2013) (26)	USA	RCT	Mean: 33.7 (10.0)	606 male	Urethritis	Gram-stained NAAT	AZT 1░g plus 14 placebo doxycycline capsules (100░mg capsules BID for 7 days) (n░=░304)		placebo AZT tablets plus 14 active doxycycline capsule (100░mg oral BID for 7 days) (n░=░302)		3, 5	3 week: AZT:%17 Doxycycline: 18 5 week: AZT: 36 Doxycycline: 39
Schwebke (2011) (27)	USA	RCT	Mean: 26.8 (6.9)	305 male	Urethritis	NAAT	AZT 1░g oral single dose (n░=░77)	AZT single 1░g oral dose plus tinidazole single 2░g oral (n░=░79)	Doxycycline 100░mg oral BID for 7 days (n░=░76)	Doxycycline 100░mg oral dose twice daily for 7 days plus tinidazole single 2░g oral (n░=░73)	2–3, 5–7	AZT arm: 22.6 Doxycycline arm: 5.2
Schillinger (2003) (28)	USA	RCT	Range: 14–34	1,787 female	Genital chlamydia	LCR or PCR	Patient-delivered partner: AZT 1░g oral single dose (patient and partner) (n░=░946)		Self-referral: AZT 1░g oral single dose (only patient) (n░=░943)		4	Patient-delivered group: 12 Self-referral group: 15
Jang (2003) (29)	Canada	RCT	Mean: 25.2 (7.1)	29 (10 men and 19 women)	Genital chlamydia	LCR, EIA, Culture	AZT 1░g oral single dose (n░=░17) 6 men and 11 women		Doxycycline 100░mg oral BID for 7 days (n░=░12) 4 men and 8 women		1, 4, 6	AZT: 17.6 Doxycycline: 0
Rustomjee (2002) (30)	South Africa	RCT	Median: 24	26 female	Cervicitis	LCR	AZT 1░g oral single dose (n░=░14)		Doxycycline 100░mg oral BID for 7 days (n░=░12)		1,2	AZT: 0 Doxycycline: 0
Skerke (2001) (31)	Croatia	RCT	Mean: 35.2 (11.02)	151 female	Urethritis	McCoy culture or by DNA/RNA digene hybridization	AZT 1░g oral single dose or AZT 500░mg once daily for 6 days (n░=░76)		Doxycycline 100░mg oral BID for 7 days or Doxycycline 100░mg oral BID for 14 days (n░=░75)		3	AZT single dose: 5.2 AZT for 6 days: 0 Doxycycline: 7 days: 5.2 Doxycycline14 days: 0
Kacmar (2001) (32)	USA	RCT	Mean: 21.4(5.7)	39 female	Genital chlamydia	LCR	AZT 1░g oral single dose (n░=░20)		500░mg amoxicillin orally three times per day for 7 days (n░=░19)		4–6	AZT: 5.26 Amoxicillin: 20
Şendaǧ (2000) - (33)	Turkey	RCT	Mean: 38.2 (1.05)	131 female	Cervicitis	Culture	AZT 1░g oral single dose (n░=░67)		Doxycycline 100░mg oral BID for 7 days (n░=░64)		2	AZT: 28.6 Doxycycline: 22.7
Hillis (1998) (34)	USA	RCT	Range: ≥ 16	196 female	Cervicitis	DFA EIA NAAT	AZT 1░g oral single dose (n░=░98)		Doxycycline 100░mg oral BID for 7 days (n░=░98)		4	AZT: 5.1 Doxycycline failure: 4.1
Thorpe (1996) (35)	USA	RCT	Mean: 24 (7)	597 female	Genital chlamydia	Clinical and a positive non-culture assay	AZT 1░g oral single dose (n░=░402)		Doxycycline 100░mg oral BID for 7 days (n░=░195)		1, 2	AZT: 2 Doxycycline: 0
Brihmer (1996) (36)	Sweden	RCT	Mean: 24.2 (7.5)	146female	Cervicitis	Culture	AZT 1░g oral single dose (n░=░72)		lymecycline 300░mg BID for 10 days (n░=░72)		2–3, 6–9	AZT: 4.34 Lymecycline: 0
Stamm (1995) (37)	USA	RCT	Mean: 26.3 (6.1)	452 male	Urethritis	Symptomatic urethritis	AZT 1░g oral single dose (n░=░248)		Doxycycline 100░mg oral BID for 7 days (n░=░123)		2, 5	AZTcumulative:19 Doxycycline cumulative: 23
Hammerschlag (1993) (38)	UK	RCT	Mean: 17.5	73 male and female	Genital chlamydia	Culture and EIA	AZT 1░g oral single dose (n░=░46)		Doxycycline 100░mg oral BID for 7 days (n░=░27)		1, 2, 4	AZT: 8.70 Doxycycline:14.80
Lauharanta (1993) (39)	Finland	RCT	Mean: 28.7 (6.7)	120 male	Urethritis	Clinical examination and culture	AZT 1░g oral single dose (n░=░60)		Doxycycline 100░mg oral BID for 7 days (n░=░60)		1, 2, 5	Follow 1: AZT: 4 Doxycycline: 0 Follow 2: AZT:10 Doxycycline: 0 Follow 3: AZT: 13 Doxycycline: 7
Martin (1992) (40)	USA	RCT	Mean: 23.5	457 (299 female and 158 male)	Genital chlamydia	Culture	AZT 1░g oral single dose (n░=░237 male: 85 female: 152)		Doxycycline 100░mg oral BID for 7 days (n░=░220 male: 73 female: 147)		1, 3, 5	AZT: 3.50 Doxycycline: 2.40
Nilsen (1992) (41)	Norway	RCT	Mean: 25.8	130 male	Urethritis	Signs and symptoms	AZT 1░g oral single dose (n░=░66)		Doxycycline 100░mg oral BID for 7 days (n░=░64)		1, 2	Follow 1: AZT: 36.40 Doxycycline: 31 Follow 2: AZT: 11.50 Doxycycline: 5.90
Whatley (1991) (42)	UK	RCT	Mean: 27.6	62 male	Urethritis	Signs and symptoms Culture	AZT 1░g oral single dose (n░=░19)	AZT 500░mg oral single dose On day 1, followed by 250░mg/day on days 2 and 3 (n░=░22)	Doxycycline 100░mg oral BID for 7 days (n░=░21)		1, 2, 4	AZT single dose: 23.50 AZT multiple dose: 5.20 Doxycycline: 16.70

RCT: Randomized controlled trial

CT: Clinical trial

AZT: Azitromycine

NAAT: Nucleic acid amplification test

PCR: Polymerase chain reaction

LCR: Ligase chain reaction

EIA: Enzyme immunoassay

DFA: Direct fluorescence assay

### 2.4 Quality assessment

The risk of bias was independently assessed by these authors using the Cochrane risk of bias assessment tool. The risks identified were compared, and disagreements were resolved by consensus. The risk of bias assessment also entailed an evaluation of the randomization method, allocation concealment, blinding of participants, researchers and assessors, incomplete outcome data, and selective outcome reporting (Table II) (21).

**Table II:** Risk of bias summary: Authors’ judgments about each risk of bias item for each included study

Domain	Selection bias	Performance bias	Detection bias	Attrition bias	Reporting bias
**Reference**					
Geisler (2015) (22)					
Geisler (2014) (23)					
Beyda (2014) (24)				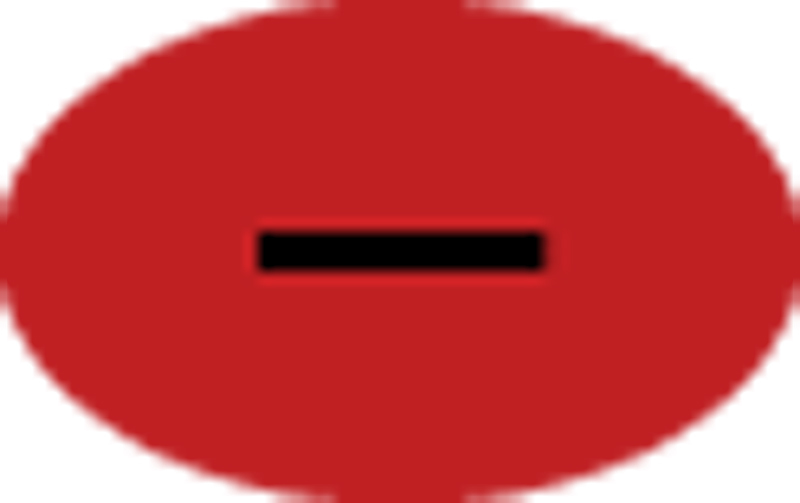	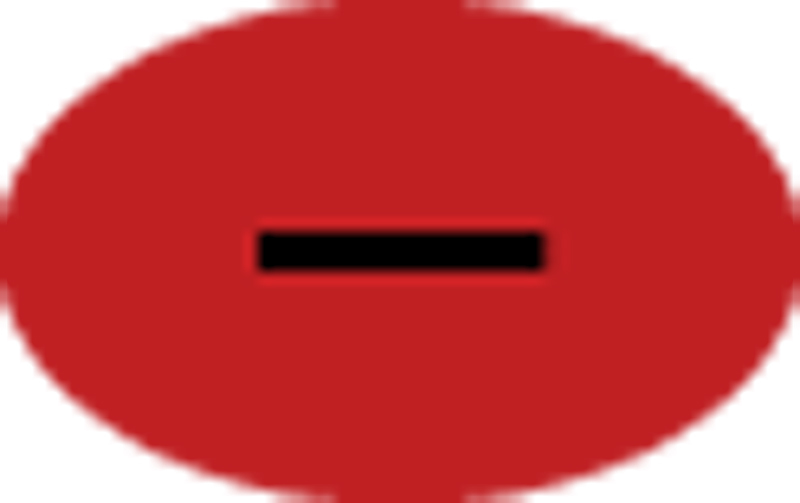
Takahashi (2014) (25)					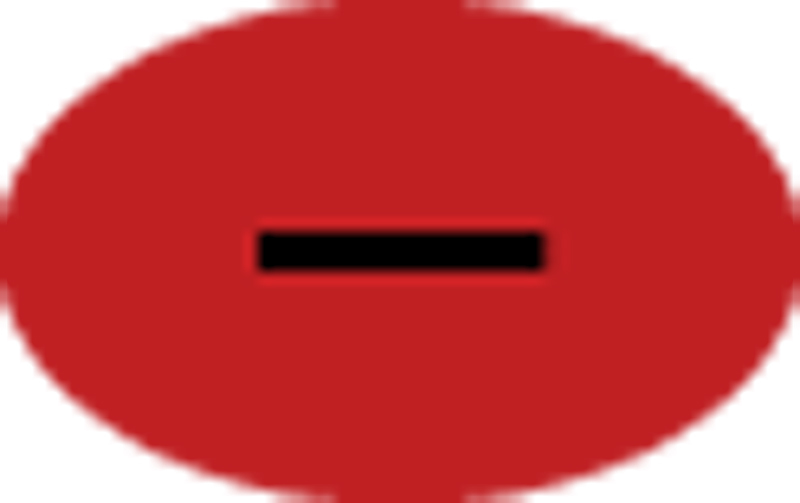
Manhart (2013) (43)					
Schwebke (2011) (27)					
Schillinger (2003)(28)					
Jang (2003) (29)	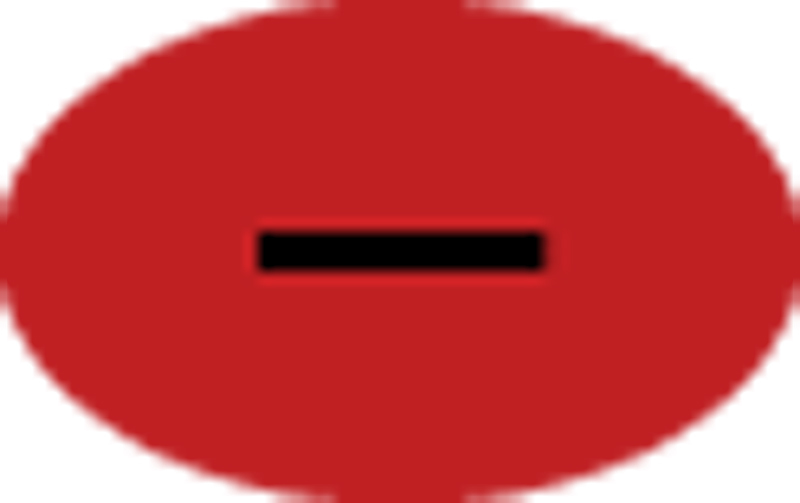				
Rustomjee (2002) (30)					
Skerke (2001) (31)					
Kacmar (2001) (32)					
Sendag (2000) - (33)					
Hillis (1998) (34)					
Thorpe (1996) (35)					
Brihmer (1996) (36)					
Stamm (1995) (37)					
Hammerschlag (1993) (38)					
Lauharanta (1993) (39)					
Martin (1992) (40)					
Nilsen (1992) (41)					
Whatley (1991) (42)					

Green░=░Low risk of bias

Red= High risk of bias

Yellow░=░Unknown bias

### 2.5 Data extraction

The first author's name, article publication year and country, study design, participants’ number and age, main complaint, diagnostic method, intervention group, the control group, follow-up period, and treatment failure were extracted and included in the analysis. Microbial recovery was defined as Chlamydia Trachomatis negativity in the biological assay (culture, enzyme immunoassay (EIA), or DNA amplification tests).

### 2.6 Statistical analyses

All the analyses were performed in Stata-14.0 (College Station, Texas) in two parts. First, the percentage of treatment failure with azithromycin in each study was extracted, and the standard error of each study was calculated using$\sqrt{\frac{p(1-P)}{n}}$, and the pooled failure rate of azithromycin was then found by inverse variance weighting. In the second part, the difference of failure rates in each study was calculated (Difference of failure rate░=░Failure rate in the intervention group - Failure rate in the control group). Next, the standard error of each study was determined using$\sqrt{\frac{p_{intervention}(1-p_{intervention})}{n_{intervention}}+\frac{p_{control}(1-p_{control})}{n_{control}}}$.

Then, the pooled difference of failure rates was obtained by inverse variance weighting. In both parts, heterogeneity was determined by Cochran's Q-Test of heterogeneity and quantified using the I^2^ index. Based on Higgins’ classification, an I^2^> 0.7 was regarded as indicative of high heterogeneity. The pooled value was estimated using the fixed-effect model, and when heterogeneity exceeded 0.7, the random-effect model was used. In both parts, the meta-regression method was used to assess the effect of age, publication year, type of study, sample size, type of disease, and follow-up as factors affecting heterogeneity among the studies. Also, Begg's test was used to investigate the publication bias; α░=░0.05 was considered as the level of statistical significance in all the analyses.

## 3 Results

The discussed stages led to the selection of 21 studies. Table I presents a full description of the selected studies. A brief description of these studies follows. The studies were all double-blind or single-blind and had open-label designs. The sample size of the selected studies varied from 26 to 1,787. A total of 6,284 people thus participated in this review study through the selected studies. The participants were of different ages and from different countries. The selected studies were RCTs (n░=░19) or CTs (n░=░2), and their follow-up periods varied from 1 to 9 wk.

The complaint of the patients with chlamydia included cervicitis (4), urethritis (8), and urogenital chlamydia (9). Chlamydia was diagnosed based on an EIA, cultures, the Nucleic-Acid Amplification Test (NAAT), Gram-stain, the Direct Fluorescence Assay (DFA), and clinical examinations, and the main outcome in all the studies was the assessment of the failure rate with azithromycin compared to other treatment strategies (i.e., Doxycycline, Rifalazil, Lymecycline, and Amoxicillin).

Since the percentage of treatment failure was reported as zero for the studies conducted by Geisler (22), Jang (23), Rustomjee (24), Skerke (25), Thorpe (26), Brihmer (27), and Lauharanta (28), 0.05 units were added to all of them in the meta-analysis to enable the inclusion of their reported results in the pooled analysis. Also, because of a high heterogeneity (I^2^ value for pooled estimate of failure rate was 94.7% (p░<░0.001), random effects method was used.

**Figure 2:**
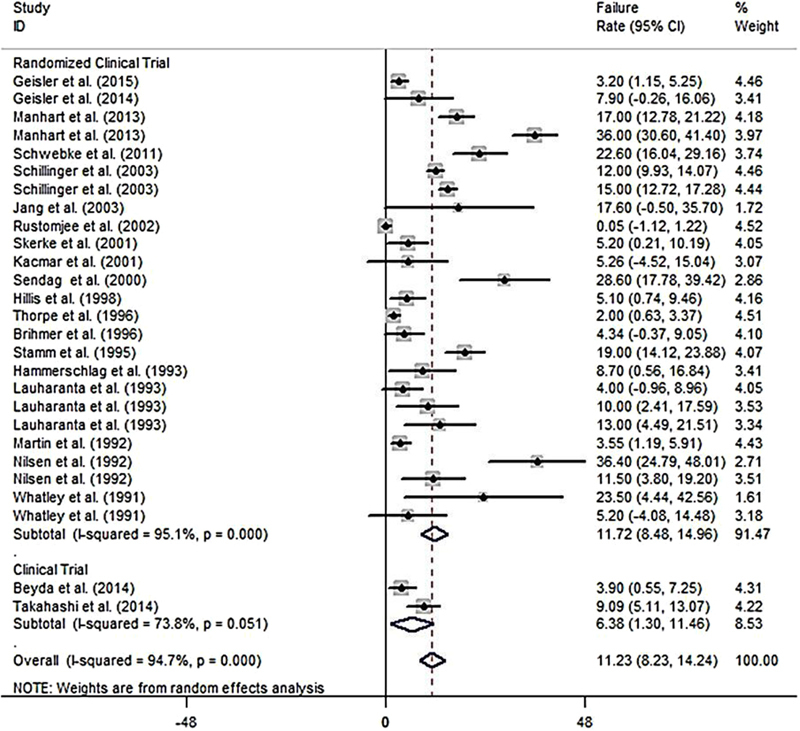
A Pooled estimate of failure rate in azithromycin user based on a random effects model in total and in different design study. The midpoint of each line segment shows the failure rate, the length of the line segment indicates the 95% confidence interval in each study, and the diamond mark illustrates the pooled failure rate.

**Figure 3:**
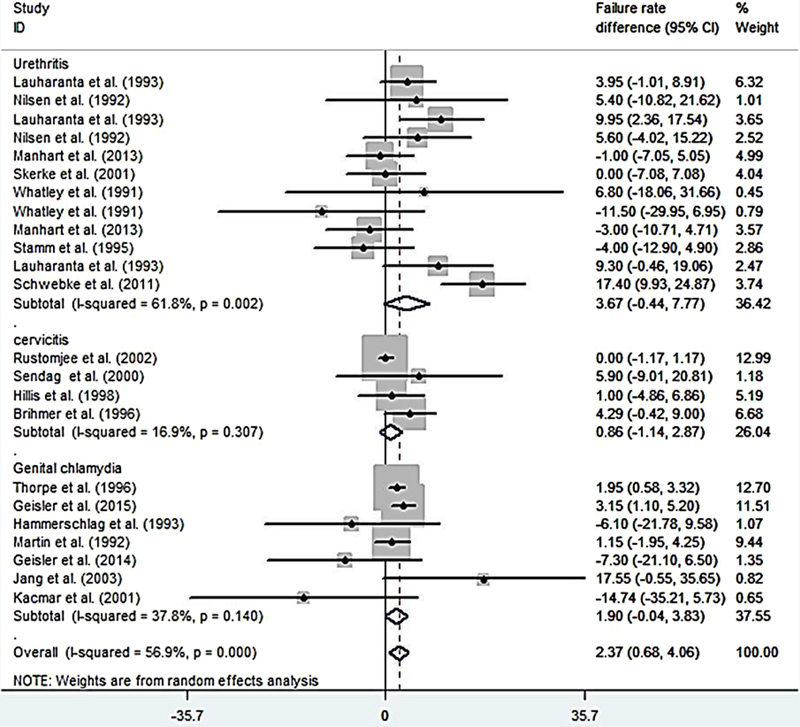
A Pooled estimate of failure rate in azithromycin user based on a random effects model in different diseases. The midpoint of each line segment shows the failure rate, the length of the line segment indicates the 95% confidence interval in each study, and the diamond mark illustrates the pooled failure rate in different design study.

The results of assessing the 21 studies showed that the pooled estimate of azithromycin failure rate was 11.23% (95% CI: 8.23%-14.24%; Figure 2), and the pooled estimate of the failure rate difference was 2.37% (95% CI: 0.68%-4.06%; Figure 3).

**Figure 4:**
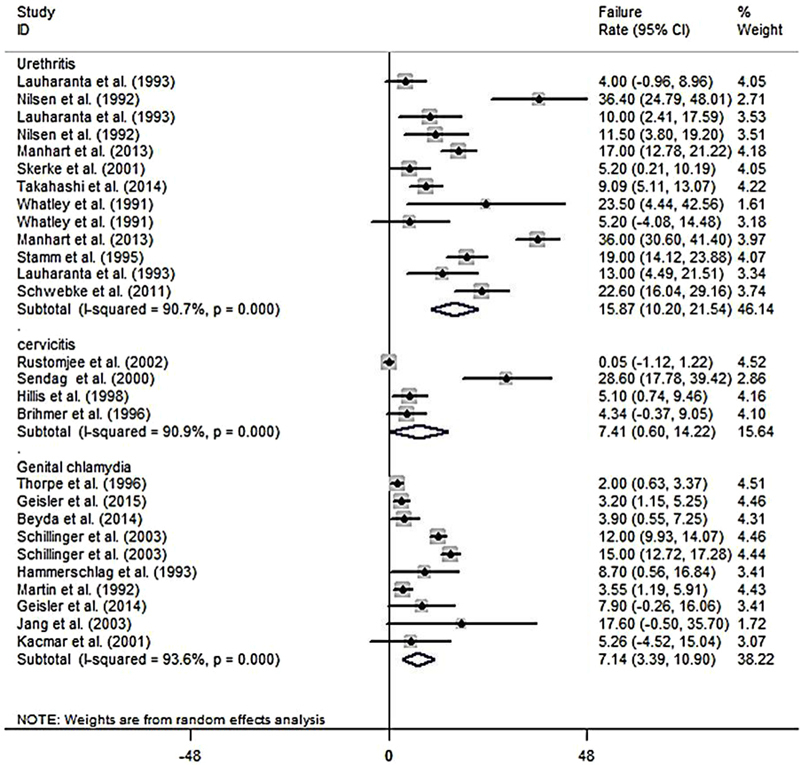
A Pooled estimate of failure rate difference (failure rate in intervention - failure rate in control) based on the random effects model in total and in different diseases. The midpoint of each line segment shows the failure rate difference, the length of the line segment indicates the 95% confidence interval in each study, and the diamond mark illustrates the pooled estimate.

In the present study, the azithromycin failure rate in the patients with chlamydial urethritis was 15.87% (CI 95%: 10.20%-21.54%; Figure 4), and the failure rate difference was 3.67% (CI 95%: -0.44%-7.77%; Figure 3). The azithromycin failure rate in the patients with chlamydial cervicitis was 7.41% (CI 95%: 0.60%-14.22%; Figure 4), and the failure rate difference was 0.86% (CI 95%: -1.14%-2.87%; Figure 3).

**Figure 5:**
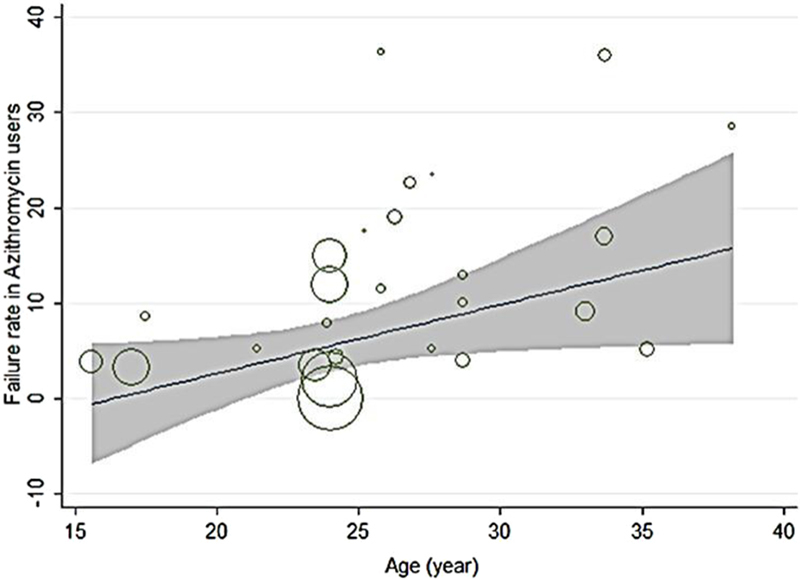
Results of meta-regression on the relation between failure rate in azithromycin user and age. The size of the circles represents the precision of each study. There is a significant relationship between failure rate and age. Failure rate relatively increased with age.

**Figure 6:**
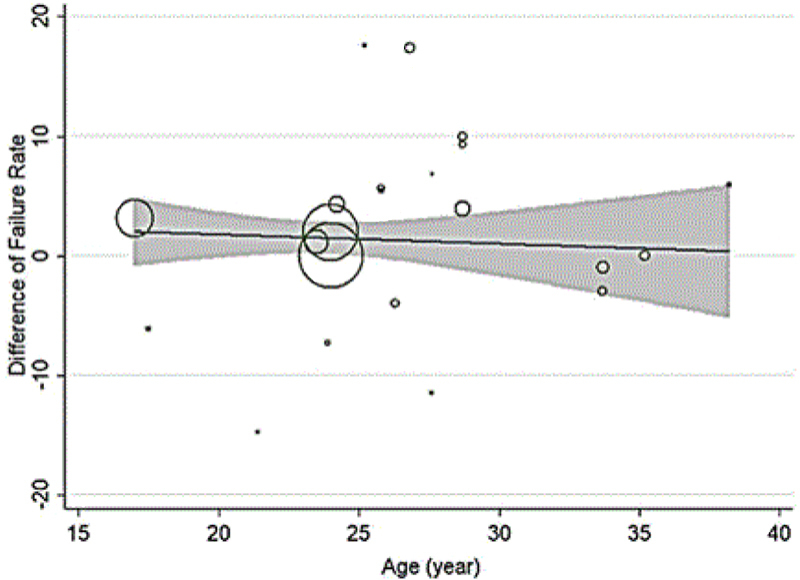
Results of meta-regression on the relation between failure rate difference and age. The size of the circles represents the precision of each study. There is no a significant relationship between failure rate difference and age.

**Figure 7:**
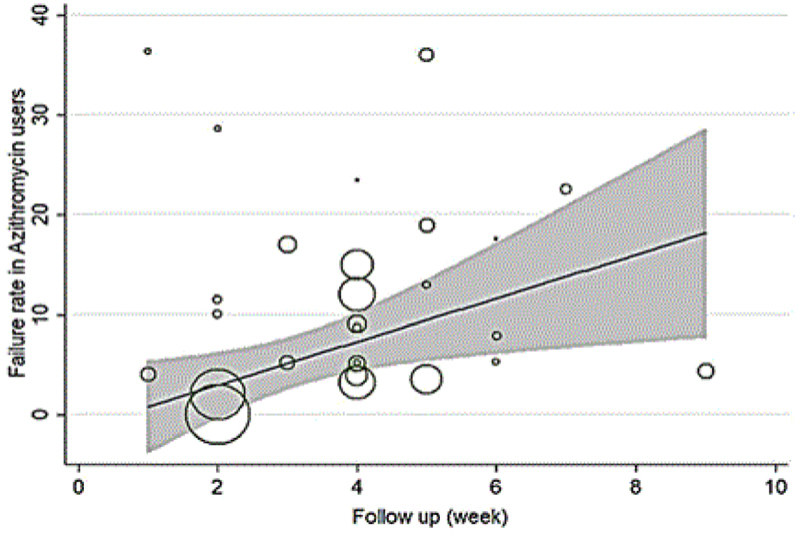
Results of meta-regression on the relation between failure rate in azithromycin user and follow up. The size of the circles represents the precision of each study. There is no a significant relationship between failure rate and follow up.

**Figure 8:**
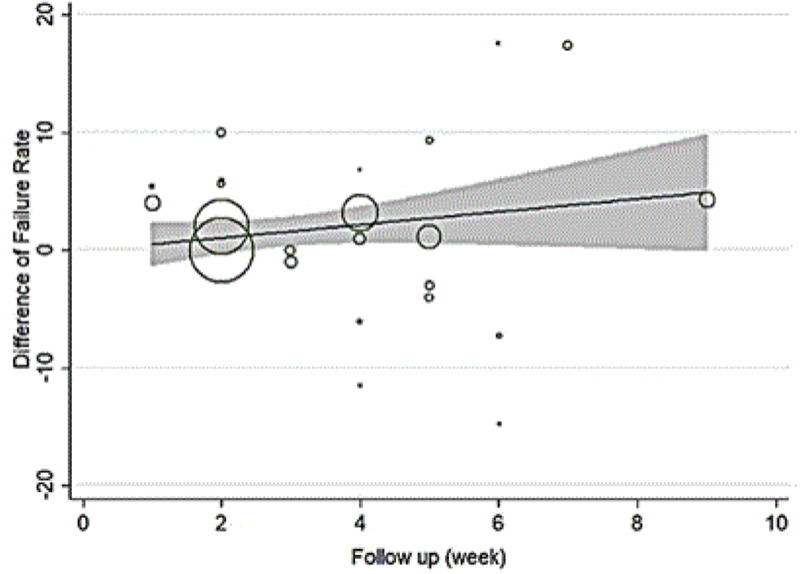
Results of meta-regression on the relation between failure rate difference and follow up. The size of the circles represents the precision of each study. There is no a significant relationship between failure rate difference and follow up.

In the patients with genital chlamydia, the azithromycin failure rate was 7.14% (CI 95%: 10.90%-3.39%; Figure 4), and the failure rate difference was 1.90% (CI 95%: -0.04%-3.83%; Figure 3). Tables III and IV show meta-regression results in order to identify the variables affecting the heterogeneity. According to this, the meta-regression results revealed that the patient's age had a significant effect on the heterogeneity in the rate of treatment failure with azithromycin (β░=░0.826; p░=░0.017; Figure 5). There is no significant relationship between failure rate difference and age (Figure 6); however, the follow-up period had no significant effect on heterogeneity in the rate of treatment failure with azithromycin (p░>░0.05; Figure 7). There is no significant relationship between failure rate difference and follow-up (p░>░0.05; Figure 8). In order to investigate the publication bias, Begg's test was used which showed that there was no publication bias (z: 1.06; p: 0.291).

**Table III:** Results of the univariate meta-regression analysis on the heterogeneity of the determinants of failure rate in azithromycin users

Variables	Coefficient	Confidence interval 95%	P-value
Age (yr)	0.826	0.159 to 1.492	0.017
Publication year (yr)	0.107	-0.359 to 0.574	0.640
Follow-up (wk)	-0.038	-2.211 to 2.135	0.971
Disease			
Cervicitis	1	-	-
Urethritis	7.422	-5.886 to 20.730	0.253
Genital chlamydia	-0.030	-4.613 to 4.553	0.989
Design			
Clinical trial	1	-	-
RCT	5.464	-8.772 to 19.701	0.437
Sample size	0.003	-0.011 to 0.019	0.510

RCT: Randomized clinical trial

**Table IV:** Results of the univariate meta-regression analysis on the heterogeneity of the determinants of different proportion of failure rate of azithromycin and other treatment strategies

Variables	Coefficient	Confidence interval 95%	p-value
Age (yr)	0.029	-0.512 to 0.572	0.910
Publication year (yr)	-0.025	-0.330 to 0.279	0.863
Follow-up (wk)	0.334	-0.822 to 1.490	0.554
Disease			
Cervicitis	1	-	-
Urethritis	1.641	-5.527 to 8.809	0.631
Genital chlamydia	0.871	-0.258 to 2.001	0.115
Sample size	-0.002	-0.014 to 0.008	0.605

## 4 Discussion

The present analysis offered a comprehensive assessment of azithromycin failure in treating patients infected with chlamydia. A number of high-quality studies with a low risk of bias were included in this assessment. Although the present findings generally confirmed the effectiveness of azithromycin in treating urogenital chlamydia, there were controversial reports of azithromycin failure rate differences.

According to the results of this meta-analysis, the azithromycin failure rate in patients infected with urogenital chlamydia was 11.23%. In one study, Kissinger and colleagues showed that the treatment failure with single 1░g dose of oral azithromycin in men with urogenital chlamydia who had sexual relations with women with chlamydial cervicitis varied from 6.2% to 12.8%, which is higher than the rate proposed by the WHO (treatment failure rate < 5%) (43). In a systematic review and meta-analysis, Kong and colleagues showed that the random-effects pooled efficacy for azithromycin (based on eight studies) was 82.9% and for doxycycline (based on five studies) was 99.6%, resulting in a random-effects pooled efficacy difference of 19.9% in favor of doxycycline. The efficacy of single-dose azithromycin was considerably lower than one week of doxycycline for treating rectal chlamydia (44).

Schillinger and colleagues reported the percentage of azithromycin treatment failure in the group where only the women underwent treatment versus the group where both women and their sexual partners underwent treatment as 15% and 12%, respectively, which means that treating the sexual partner can help prevent the recurrence of chlamydia infection (28). Kong and colleagues compared the effectiveness of single 1░g dose of oral azithromycin with single 1░g░r dose of azithromycin plus twice daily 100░mg doses of doxycycline over seven days in men with rectal chlamydia and reported failure rates of 12.8% and 0% in the two groups, respectively (45). In a meta-analysis conducted to determine and compare the effectiveness of azithromycin and doxycycline in treating genital chlamydia, Kong and colleagues reported failure rates of 4% and 3% for azithromycin and doxycycline, respectively. The subgroup analysis showed that the fixed and random effects of the pooled efficacy difference in symptomatic men were 4.7% and 5.5%, respectively (46).

The present study revealed a failure rate difference of 2.37%, which suggests that the rate of treatment failure is higher with azithromycin than with doxycycline and other examined medications in treating chlamydia. In an in vitro study to investigate the sensitivity of Chlamydiatrachomatis strains, Ljubin and colleagues reported that all the strains showed sensitivity to azithromycin and doxycycline with a Minimum Inhibitory Concentration (MIC) (47). Bhengraj and colleagues used cell cultures to extract 21 clinical isolates from women with symptoms of frequent chlamydia infection and administered azithromycin and doxycycline with MICs ≤ 0.125 and ≤ 0.25░µg/ml, respectively. They reported that 38% of the samples did not respond to the MIC, and a reduction was observed in sensitivity to the current first-line antibiotics (azithromycin and doxycycline) for the treatment of chlamydia infections in isolates taken from patients with recurring infection (48).

Schwebke and colleagues investigated the effects of single 1░g dose of azithromycin and twice daily 100░mg doses of doxycycline over seven days in patients with chlamydia urethritis, and also the effect of adding 2░g of tinidazole to these treatment regimens. They found that the addition of tinidazole to the treatment regimen does not increase the rate of recovery but eradicates chlamydia effectively. The difference in clinical recovery between the groups was not significant; however, doxycycline had considerably better efficacy than azithromycin, and the treatment failure was reported as 22.6% in the azithromycin group and 5.2% in the doxycycline group (27).

In the present study, the azithromycin failure rate was 15.85% and the failure rate difference was 3.67% in patients with chlamydial urethritis. In their three follow-ups of men with chlamydia urethritis undergoing clinical examinations and urinary tract culture, Lauharanta and colleagues reported the failure rates in the first, second, and fifth weeks of the follow-up as 4%, 10%, and 13% in the group receiving single 1░g dose of azithromycin, and as 0%, 0%, and 7% in the group receiving twice daily 100░mg doses of doxycycline over seven days, and no significant differences were thus observed between the two groups (39).

In a study conducted by Nilsen and colleagues, treatment failure in the azithromycin and doxycycline groups in the first and second weeks of the follow-up were reported as 36.40% versus 32% and 11.5% versus 5.9%. These researchers reported no significant differences in the treatment failure rate between the two groups (41). In a clinical trial on 606 men with chlamydia urethritis, Manhart and colleagues compared the effects of a single 1░g dose of azithromycin and twice daily 100░mg doses of doxycycline over seven days, and showed a fairly poor clinical and microbiological recovery rates in both groups, with no significant differences observed between azithromycin and doxycycline, as the treatment failure rates in the third and fifth weeks of the follow-up were 17% and 36%, and 18% and 39%, respectively (26).

Skerk and colleagues reported the treatment failure rates of single 1░g dose of azithromycin, once daily 500░mg dose of azithromycin over six days, twice daily 100░mg doses of doxycycline over seven days, and twice daily 100░mg doses of doxycycline over 14 days as 5.2%, 0%, 5.2%, and 0%, respectively (31). In a study conducted by Takahashi and colleagues in Japan to determine the clinical effect of single 2░g dose of oral azithromycin in men with chlamydia urethritis, the treatment failure rate was 9.09% (25). In a study by Whatley and colleagues, men diagnosed with chlamydia urethritis were treated with single 1░g dose of oral azithromycin, single 500░mg dose of oral azithromycin on the first day and 250░mg oral azithromycin on the 2nd and 3rd days, or twice daily 100░mg doses of doxycycline over seven days. The treatment failure rates with a single dose of azithromycin and multiple doses of azithromycin and doxycycline groups were reported as 23.5%, 5.20%, and 16.70%, respectively. All three regimens were well tolerated by the patients, but the single and multiple doses of azithromycin showed better therapeutic effects than doxycycline (42).

In their study, Stamm and colleagues compared the effects of single 1░g dose of oral azithromycin and twice daily 100░mg doses of doxycycline over seven days in men with chlamydial urethritis and reported treatment failure rates of 19% for azithromycin and 23% for doxycycline (37). Deguchi and colleagues investigated the therapeutic effects of a single 2░g dose of oral azithromycin and twice daily 100░mg doses of sitafloxacin over seven days in men with chlamydial urethritis. In their nine-week follow-up, they showed treatment failure rates of 4% in the azithromycin group and 0% in the sitafloxacingroup and recommended both azithromycin and sitafloxacin as treatments for chlamydia infections (49).

The present study showed a failure rate of 7.41% (CI 95%: 0.60%-14.22%) for azithromycin and a failure rate difference of 0.86 (CI 95%: -1.14%-2.87%) in patients with chlamydial cervicitis. Rustomjee and colleagues compared the therapeutic effects of single 1░g dose of azithromycin and twice daily 100░mg doses of doxycycline over seven days in 26 women with chlamydial cervicitis and showed a similar failure rate of 0% in both groups (30). In their study, Sendag and colleagues compared the therapeutic effects of a single 1░g dose of azithromycin and twice daily 100░mg doses of doxycycline over seven days in women with non-gonococcalmucopurulentendocervicitis. Two weeks after the treatment, the infection eradication rate was reported as 71.4% in the azithromycin group and 77.3% in the doxycycline group, with no statistically significant differences between the single dose of azithromycin and oral doxycycline (33).

Hillis and colleagues conducted a clinical trial in women with cervicitis to determine the therapeutic effects of single 1-g dose of azithromycin and twice daily 100░mg doses of doxycycline over seven days in preventing persistent or recurring chlamydia over one month, and reported a treatment failure of 5.1% in the azithromycin group and 4.1% in the doxycycline group (34). Brihmer and colleagues compared women with chlamydial cervicitis in terms of the therapeutic effects of a single 1░g░r oral dose of azithromycin and twice daily 300░mg doses of lymecycline over seven days and reported a treatment failure rate of 4.34% and 0% for azithromycin and lymecycline, respectively (36). Lowrence and colleagues investigated the recovery rate with 1░g of azithromycin in six strains clinically isolated through culture and PCR and reported no genomic mutations in the ribosomal leukocytes that could develop a direct resistance to azithromycin (50).

In an in vivo study, Yeruva and colleagues investigated the therapeutic effects of azithromycin on the chlamydial infection of the gastrointestinal tract and the cervix, and 10 eight-week-old rats were administered a single oral dose of azithromycin 10 days after the infection at three different doses, that is, 20, 40, and 80░mg/kg, or an intraperitoneal injection of 10░mg/kg of doxycycline over seven days. They reported that the antibiotic levels were sufficient for the treatment of cervical infection and all the rats receiving the three different doses of azithromycin or doxycycline recovered from the infections, but the treatment regimens were ineffective on gastrointestinal infections, and further antibiotic assessments are needed for chlamydial infections (51). Ossewaard and colleagues compared the efficacy of single 1░g dose of azithromycin with twice daily 100░mg doses of doxycycline over the standard period of seven days in treating cervical infections caused by Chlamydiatrachomatis. The culture of all the samples taken from both groups one and four weeks after the initiation of treatment were negative (52).

In the present study, the azithromycin failure rate was 7.14% (CI 95%: 3.39%-10.9%) and the failure rate difference was 1.90% (CI 95%: -0.04%-3.83%) in patients with genital chlamydia. Thorpe and colleagues compared the therapeutic effects of a single 1░g oral dose of azithromycin and twice daily 100░mg doses of doxycycline over seven days in women with urethritis or cervicitis and reported treatment failure rates of 2% and 0%, respectively (35). Geisler and colleagues studied men and women with urogenital chlamydia who were randomly assigned to a single-dose 1░g azithromycin group or a twice daily 100░mg dose of doxycycline group over seven days. Azithromycin was 97% effective and doxycycline 100% (22). Beyda and colleagues also investigated the effectiveness of azithromycin in adolescent girls and boys with cervicitis or urethritis caused by chlamydiatrachomatisinfection and reported the effectiveness of azithromycin in treating chlamydia as 96.1% (24).

Hammerschlag and colleagues studied women and men with clinical chlamydial cervicitis and urethritis and reported treatment failures of 8.70% and 14.80% for the single 1░g oral dose of azithromycin and twice daily 100░mg doses of doxycycline over seven days (38). Martin and colleagues compared the therapeutic effects of a single 1░g oral dose of azithromycin and twice daily 100░mg doses of doxycycline over seven days in women and men with chlamydial cervicitis or urethritis and reported a treatment failure of 3.50% for azithromycin and 2.40% for doxycycline (40). Geisler and colleagues reported the treatment failure rates of 15.2% with a single 25░mg dose of Rifalazil and 7.9% with a single 1░g dose of azithromycin in women with genital chlamydia (23).

Jang and colleagues reported treatment failures of azithromycin and doxycycline as 17.6% and 0%, respectively, in women with genital chlamydial infections (29). Kacmar and colleagues conducted a randomized clinical trial in patients with genital chlamydia with a single 1░g dose of azithromycin versus thrice daily 500░mg doses of amoxicillin over seven days and reported the treatment failure rates of azithromycin and amoxicillin as 5.26% and 20%, respectively (32).

In one study, Quinn and colleagues stated that, based on the recent recommendation of the Center for Disease Control and Prevention (CDC) regarding the high efficacy of azithromycin and doxycycline (97% and 100%) in treating genital chlamydia, these medications are valid and appropriate (53). In a prospective cohort study to determine the recovery rate of anorectal and cervicovaginal chlamydia following treatment with a single 1░g oral dose of azithromycin, Dukers and colleagues reported a treatment failure of 15–27% over the three-week and eight-week follow-ups after the initiation of the treatment (54).

In another study, Tan and colleagues compared the therapeutic effects of a single 1░g oral dose of azithromycin and twice daily 100░mg doses of doxycycline over seven days in treating men with non-gonococcal urethritis and also female sex workers with chlamydial cervicitis. Both medications were highly successful in eradicating proven chlamydial infections, and the treatment success rate was 100% in men with NGU, and 96.6% for azithromycin and 100% for doxycycline in the cases of chlamydial cervicitis (55). In a clinical trial conducted to assess the efficacy of azithromycin, Steingrimsson and colleagues stated that 96% of patients with genital chlamydia were cured with azithromycin (56). Importantly, despite the reports of a high failure rate with 1░g doses of azithromycin in the reviewed studies, only two studies were found on the effect of azithromycin on rectal chlamydia; however, they did not satisfy our inclusion criteria. Given the potential comorbidity of HIV and rectal chlamydial infection, it is imperative that effective treatments be available (Bernstein and colleagues). Given these concerns, the current European guidelines recommend that rectal chlamydial infection should be treated with doxycycline over seven days (57). Nonetheless, further clinical trials in this area seem necessary.

As can be seen, the results of various studies on the treatment failure of therapeutic regimens are different in the treatment of urogenital chlamydia trachomatis, which can be due to the difference in the prevalence of the disease due to the cultural and social conditions in different societies as well as due to the differences in the type of treatment and duration of treatment. According to the WHO guidelines, those treatment regimens are recommended for sexually transmitted infections that present efficacy of more than 95% (58). Given the present findings, policymakers should decide whether a change is necessary for the guidelines or not. Previous studies have shown that patients with chlamydial infection symptoms have high organism loads, which could be associated with azithromycin treatment failure (59,60). Given the high failure rate of single-dose oral azithromycin in treating chlamydial infections and the increasing concerns about the serious complications caused by the poor-to-moderate efficacy of azithromycin, well-designed double-blind studies with high statistical power are required on this subject.

### 4.1 Limitations of the study

Some studies that met the inclusion criteria of the present study had been omitted from the databases. Also, some of the recent studies on azithromycin failure in treating chlamydia infections were only accessible via their abstracts.

## 5 Conclusion

In the 21 reviewed clinical trials, the azithromycin treatment failure rate was 11.23% and the failure rate difference was 2.37%. Azithromycin has a higher failure rate than doxycycline and other examined medications in treating urogenital chlamydia infections. Given that the treatment failure rate with azithromycin is higher than that expected by the WHO for the treatment of sexually transmitted infections (under 5%), more well-designed studies are needed to assess the therapeutic effects of other medications on chlamydia infections.

## Conflict of Interest

None of the authors have a conflict of interest.
